# A Green Fluorescent Protein Containing a QFG Tri-Peptide Chromophore: Optical Properties and X-Ray Crystal Structure

**DOI:** 10.1371/journal.pone.0047331

**Published:** 2012-10-10

**Authors:** Jion M. Battad, Daouda A. K. Traore, Emma Byres, Jamie Rossjohn, Rodney J. Devenish, Seth Olsen, Matthew C. J. Wilce, Mark Prescott

**Affiliations:** 1 Department of Biochemistry and Molecular Biology, School of Biomedical Sciences, Monash University, Clayton, Victoria, Australia; 2 The Structural Biology Unit, Department of Biochemistry and Molecular Biology, School of Biomedical Sciences, Monash University, Clayton, Victoria, Australia; 3 School of Mathematics and Physics, The University of Queensland, Brisbane, Queensland, Australia; Cardiff University, United Kingdom

## Abstract

Rtms5 is an deep blue weakly fluorescent GFP-like protein (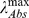, 592 nm; 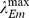, 630nm; Φ_F_, 0.004) that contains a ^66^Gln-Tyr-Gly chromophore tripeptide sequence. We investigated the optical properties and structure of two variants, Rtms5^Y67F^ and Rtms5^Y67F/H146S^ in which the tyrosine at position 67 was substituted by a phenylalanine. Compared to the parent proteins the optical spectra for these new variants were significantly blue-shifted. Rtms5^Y67F^ spectra were characterised by two absorbing species (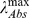, 440 nm and 513 nm) and green fluorescence emission (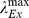, 440 nm; 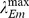, 508 nm; Φ_F_, 0.11), whilst Rtms5^Y67F/H146S^ spectra were characterised by a single absorbing species (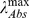, 440 nm) and a relatively high fluorescence quantum yield (Φ_F,_ 0.75; 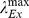, 440 nm; 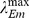, 508 nm). The fluorescence emissions of each variant were remarkably stable over a wide range of pH (3–11). These are the first GFP-like proteins with green emissions (500–520 nm) that do not have a tyrosine at position 67. The X-ray crystal structure of each protein was determined to 2.2 Å resolution and showed that the benzylidine ring of the chromophore, similar to the 4-hydroxybenzylidine ring of the Rtms5 parent, is non-coplanar and in the *trans* conformation. The results of chemical quantum calculations together with the structural data suggested that the 513 nm absorbing species in Rtms5^Y67F^ results from an unusual form of the chromophore protonated at the acylimine oxygen. These are the first X-ray crystal structures for fluorescent proteins with a functional chromophore containing a phenylalanine at position 67.

## Introduction

GFP-like proteins are valuable tools for use in molecular cell biology applications [1], [2]. Extensive engineering has resulted in a range of proteins whose fluorescence emissions extend over the entire visible range. Many of the proteins have been cloned and developed from a limited number of naturally occurring fluorescent progenitors that include *Aequorea victoria* GFP (*av*GFP) [3] and DsRed isolated from *Discosoma* species [4]. Some non-fluorescent proteins such as hcCP, a chromoprotein isolated from *Heteractis crispa* have served as a valuable source of far-red fluorescent proteins that include HcRed [5].

Formation of the chromophore in GFP-like proteins is the result of a series of post-translational autocatalytic events involving a tri-peptide motif. All naturally occurring GFP-like proteins isolated to date contain the tri-peptide X-Tyr-Gly, however the tyrosine can be substituted for other amino acids resulting in proteins with different optical properties. For example, substituting the chromophore tyrosine in *av*GFP with tryptophan or histidine resulted in blue-shifted fluorescent proteins (FPs) with cyan and blue fluorescence emissions, respectively [6]. A phenylalanine substitution results in FPs with the most blue-shifted emissions such as the *av*GFP^Y66F^ (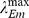, 442 nm) [7], and the more recently developed Sirius (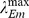, 424 nm) [8].

A number of covalent modifications have been identified that further expand the range of optical properties including alternative chromophore structures [9]. For example, the red-shifted optical characteristics of DsRed and eqFP611 are the result of an acylimine linkage extending the chromophore conjugation system [10], [11]. In addition to providing the appropriate environment to promote chromophore formation, contacts between the mature chromophore and the protein matrix determine the optical properties of these proteins. For instance, a Thr203Tyr substitution introduced to *av*GFP resulted in the first yellow fluorescent protein [12], whilst contacts with the acylimine oxygen are believed to contribute to the red-shifted properties of mPlum and Neptune [13], [14].

Rtms5 is a deep blue weakly fluorescent GFP-like protein (Φ_F_, 0.004; 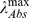, 592 nm) isolated from the coral *Montipora efflorescens* [15]. The X-ray crystal structure of Rtms5 suggests that its low fluorescence emission results from the *trans* non-coplanar configuration of the chromophore derived from an Gln-Tyr-Gly tripeptide [15]. An Rtms5^H146S^ variant was significantly more fluorescent than Rtms5 particularly at high pH (Φ_F_, 0.16 at pH 11.0; 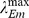, 630 nm), and the X-ray crystal structure showed evidence for a chromophore in a *cis*-coplanar configuration [16]. The chromophore in Rtms5 is extended by the presence of an acylimine linkage, and is in part responsible for the red-shifted optical properties of this protein [15]–[18].

Remarkably, there are few reports in the literature describing the properties of FPs with a phenylalanine in the chromophore tripeptide (i.e. X-Phe-Gly), and no X-ray crystal structures are available, other than those for proteins that do not have a correctly formed GFP-like chromophore [19]. Therefore, in this study we set out to investigate the optical properties and structure of Rtms5 and Rtms5^H146S^ each containing a Tyr67Phe substitution. The resulting proteins, Rtms5^Y67F^ and Rtms5^Y67F/H146S^, have green fluorescence emission (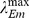, 508 nm), and are the first FPs reported that have both green emissions (500–525 nm) and a phenylalanine in the chromophore tripeptide. The X-ray crystal structure of each of the variants was determined to 2.2 Å resolution. The structures show evidence for the presence of an acylimine linkage extending the chromophore conjugation system that contributed to the green fluorescence emission. The chromophores are in a *trans* non-coplanar conformation. To our knowledge, these are the first reported X-ray structures for GFP-like proteins containing a functional phenylalanine-substituted chromophore.

## Results

### Optical Properties of Rtms5^Y67F^ and Rtms5^Y67F/H146S^

**Figure 1:**
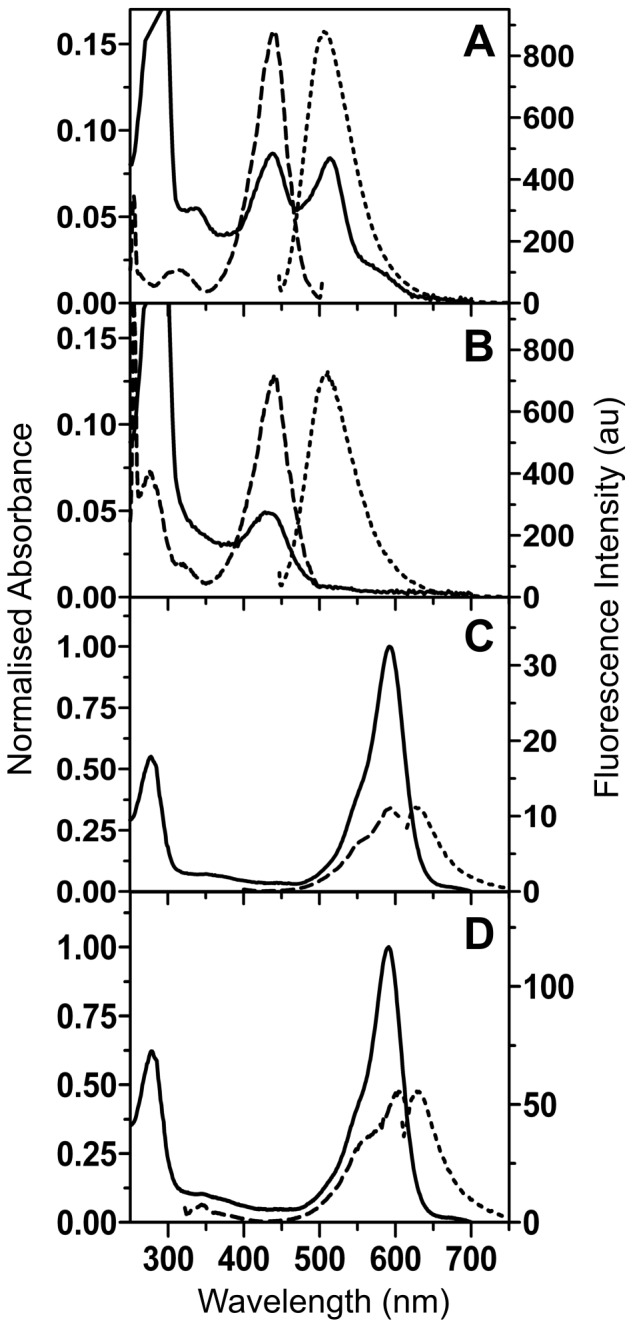
Absorbance and fluorescence spectra for Rtms5^Y67F^, Rtms5^Y67F/H146S^, Rtms5 and Rtms5^H146S^. Spectra for Rtms5^Y67F^, (A); Rtms5^Y67F/H146S^, (B); Rtms5, (C) and Rtms5^H146S^, (D) were determined in 20 mM Tris-HCl, pH8.0 and 300 mM NaCl. Absorbance spectra are normalised at 280 nm. Absorbance (solid line), excitation (dashed line), and emission (dotted line).

In order to investigate the effects of a tyrosine to phenyalanine substitution in Rtms5 and Rtms5^H146S^ we determined the absorbance and fluorescence spectra for Rtms5^Y67F^ and Rtms5^Y67F/H146S^ at pH 8.0, and compared them to Rtms5 and Rtms5^H146S^, the parent proteins from which they were derived [15]. The absorbance spectrum for Rtms5^Y67F/H146S^ showed a single species (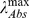, 430 nm) whilst the absorbance spectrum for Rtms5^Y67F^ showed two major species (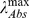, 440 nm and 513 nm) and a shoulder at ∼589 nm (Fig. 1a and b). The fluorescence excitation and emission spectra for Rtms5^Y67F^ and Rtms5^Y67F/H146S^ were similar (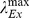, 440 nm; 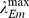, 508 nm) (Fig. 1), but compared to Rtms5^Y67F^ (Φ_F_, 0.11) the fluorescence quantum yield for Rtms5^Y67F/H146S^ (Φ_F_, 0.75) was somewhat higher. No significant florescence emission was observed when the 513 nm species of Rtms5^Y67F^ was excited. By comparison the tyrosine-containing chromophores of Rtms5 and Rtms5^H146S^ show a single red-shifted absorbing species (Fig 1c and d; 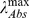, 592 nm and 588 nm, respectively) and very weak fluorescence emissions (Φ_F,_ 0.004 and 0.02 for Rtms5 and Rtms5^H146S^, respectively). The optical characteristics determined for proteins in this study are summarised and compared to those of other selected proteins in Table 1. Collectively these data indicate that a Tyr to Phe substitution results in Rtms5 variants that have significant blue-shifts in their optical spectra (∼150 nm in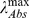), and a significant increase in Φ_F_.

**Table 1:** Optical properties of Rtms5 variants and selected fluorescent proteins.

Protein	Chromophore Tripeptide	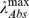 _ (nm)_	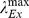 _ (nm)_	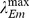 _ (nm)_	ε(M^−1^cm^−1^)	ΦF	pKa	Brightness*
Rtms5^Y67F^	QFG	440/513	440	508	3,000/2,900	0.11	<3	0.3
Rtms5^Y67F/H146S^	QFG	440	440	508	1,600	0.75	<3	1.2
mBlueberry2 [24]	MFG	402	402	467	51,000	0.48	<2.5	5.3
Sirius [8]	QFG	355	355	424	15,000	0.24	<3	3.6
Rtms5 [15]	QYG	592	592	626	80,000	0.004	3.2	0.3
Rtms5^H146S^ [15]	QYG	588	602	628	80,000	0.02	4.6	1.6
Rtms5^H146S^ at pH11 [16]	QYG	582	570	620	62,000	0.16	4.6	9.9

* Brightness calculated using ε*Φ_F_/1000.

**Figure 2:**
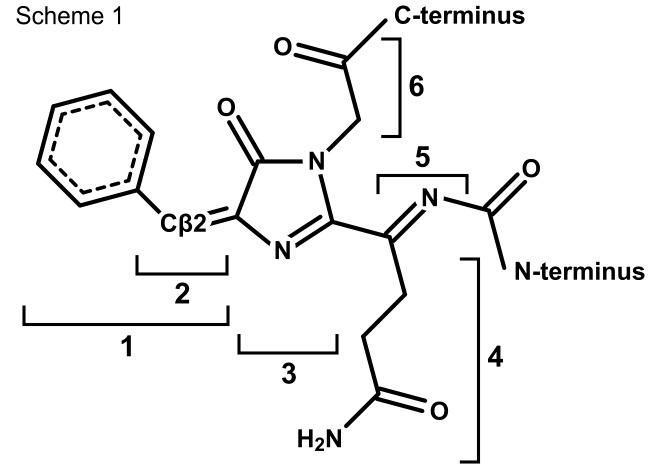
Chemical structure of the chromophore in Rtms5^Y67F^ and Rtms5^Y67F/H146S^. The chemical structure of the mature chromophore is shown. Individual moieties identified in the text are labelled: (1), benzylidine; (2), methine; (3), imidazalinone; (4) glutaminyl; (5), acylimine linkage; and (6) glycyl. The location of the N- and C-termini are indicated.

**Figure 3:**
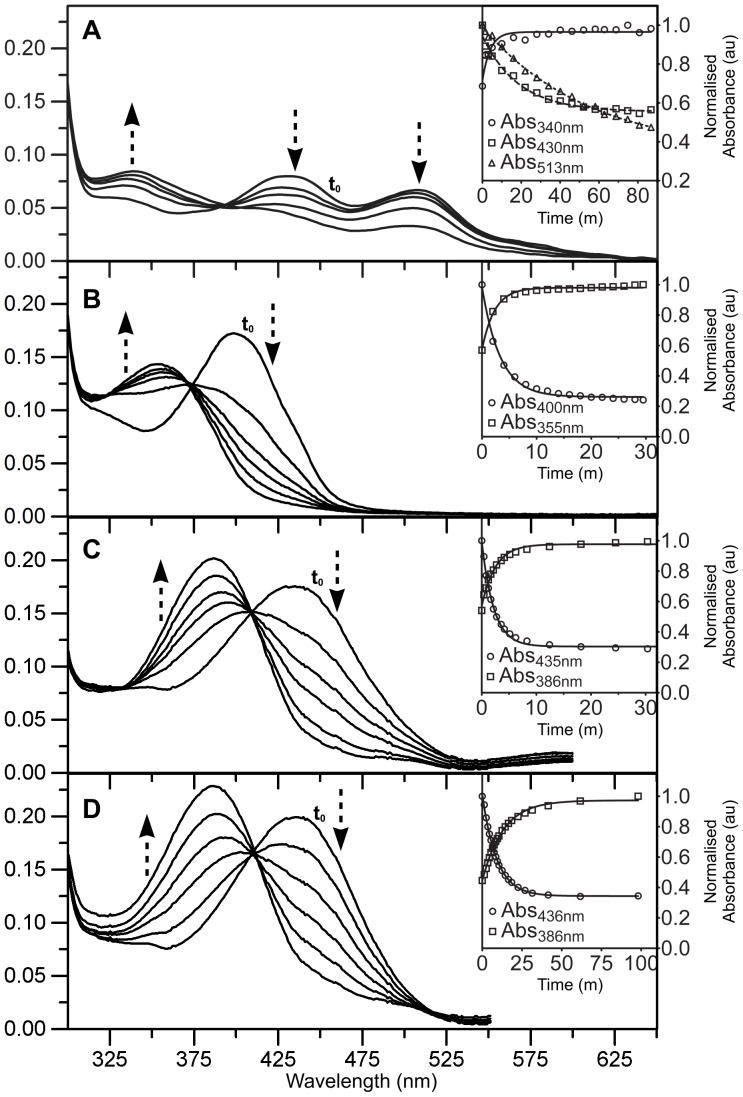
The effect of low pH on the absorbance spectra of Rtms5^Y67F^ and Rtms5^Y67F/H146S^. Rtms5^Y67F^ (A) and Rtms5^Y67F/H146S^ (B) at a protein concentration of 0.25 mg/ml in 0.1 M potassium phosphate, pH 2.3 were incubated at 21°C and the absorption spectra determined at selected time points. Rtms5 (C) and Rtms5^H146S^ (D) at a protein concentration of 0.30 mg/ml in 0.1 M potassium phosphate, pH 2.3 were included as controls. The first absorbance scan of the incubation mixture (t_0_) is indicated. Relative trends (decrease or increase) in the absorbance spectra at different positions are indicated by arrows. The kinetics for changes in amount of individual absorbing species for each protein are shown (inset).

Interestingly, compared to the phenylalanine-substituted chromophore of Sirius (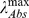, 355 nm), a blue-emitting FP derived from *av*GFP, the chromophores of Rtms5^Y67F^ and Rtms5^Y67F/H146S^ are red-shifted by ∼ 86 nm. Since the Rtms5 and Rtms5^H146S^ chromophores are reported to contain an acylimine linkage (Fig. 2) that extends their conjugation system and contributes to their red-shifted optical properties [15], we were prompted to investigate the possibility that the Rtms5^Y67F^ and Rtms5^Y67F/H146S^ chromophores also contained an acylimine linkage. Acylimine linkages are susceptible to nucleophilic attack, and when present in FPs undergo addition of water across the double bond when the protein is exposed to extremes of pH. Acylimine hydration results in a reduction in the extent of conjugation of the chromophore, and a characteristic blue-shift in its absorbance spectra [10], [17], [20]. Rtms5^Y67F^ or Rtms5^Y67F/H146S^ were incubated in buffer at pH 2.3, and their absorbance spectra determined at selected time points. The acylimine-containing chromophores of Rtms5 and Rtms5^H146S^ were included in this study as positive controls [15]. The results show that incubation of Rtms5^Y67F/H146S^ led to a decrease in the amount of the 400 nm species and a corresponding increase in the amount of a 355 nm species. A single isosbestic point at 375 nm was observed indicating that these two species are stoichiometrically related (Fig. 3b). The blue-shift in the Rtms5^Y67F/H146S^ absorbance spectrum indicates a reduction in the extent of chromophore conjugation resulting from hydration of an acylimine linkage. The control proteins Rtms5 and Rtms5^H146S^ which are known to contain an acylimine linkage [15], [18] undergo a characteristic blue-shift (435 nm to 386 nm) in their absorbance spectra with an isosbestic point at 410 nm (Fig. 3c and d). Collectively, these data indicate that the Rtms5^Y67F/H146S^ chromophore contains an acylimine linkage.

Changes in the absorbance spectrum for Rtms5^Y67F^ incubated at pH 2.3 appeared more complex (Fig. 3a). At low pH a decrease in amounts of the 425 nm and 513 nm species was associated with a corresponding increase in the amount of the 349 nm species. These changes were irreversible as the 425 and 513 nm species did not reappear when the reaction mixture from the end point of the reaction was titrated back to pH 8.0. These results suggest that both the 513 nm and 425 nm chromophore species contain an acylimine linkage. The presence of a single isosbestic point at 390 nm suggests that both the 425 nm and 513 nm species exchange with the 349 nm species. The absence of a clear isosbestic point between the 425 nm and 513 nm species suggests that the 513 nm exchanges with the 390 nm independently of the 425 nm species.

**Figure 4:**
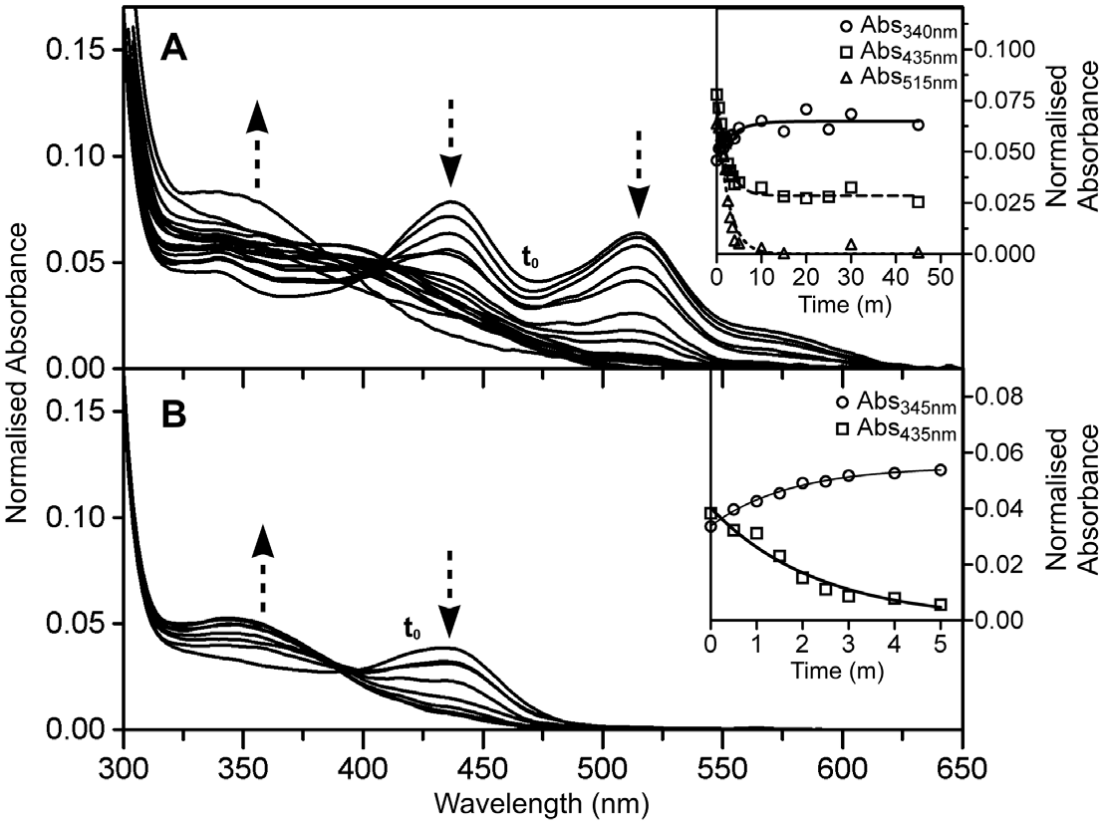
The effect of GuHCl on the absorbance spectra of Rtms5^Y67F^ and Rtms5^Y67F/H146S^. Rtms5^Y67F^ (A) and Rtms5^Y67F/H146S^ (B) were diluted to a final protein concentration of 0.15 mg/ml in 0.1 M Tris-HCl (pH 8.0), 6 M GuHCl, and the absorption spectra determined at selected time points after incubation at 21°C. The first absorbance scan (t_0_) is indicated. Relative trends (decrease or increase) in absorbance are indicated by arrows. The kinetics for individual absorbing species is shown for each protein (inset).

In order to help exclude the possibility that exposure of proteins to low pH contributed to some change in chromophore structure, other than hydrolysis of the acylimine linkage, we investigated the chromophore at pH 8.0 in the presence of a protein denaturant. Guanidine HCl (GuHCl) promotes protein unfolding thereby exposing the chromophore acylimine linkage to the bulk solvent, and subsequent nucleophilic attack and hydration. We incubated Rtms5^Y67F^ and Rtms5^Y67F/H146S^ in 6 M GuHCl at pH 8.0, and determined the absorbance spectra at selected time points. For Rtms5^Y67F^ the amounts of the 515 nm and 453 species decreased, leading to a corresponding increase in the 345 nm species (Fig. 4a). For Rtms5^Y67F/H146S^ the amount of the 435 nm and 340 nm species decreased and increased, respectively (Fig. 4b). Collectively, these results together with those obtained at low pH suggest that all chromophore species in these proteins contain an acylimine linkage, and that the 513 nm species of Rtms5^Y67F^ likely arises from alternate interactions of the chromophore with the protein matrix, and not a separate covalent modification of the Rtms5^Y67F^chromophore. Structural evidence presented later supports such a possibility.

**Figure 5:**
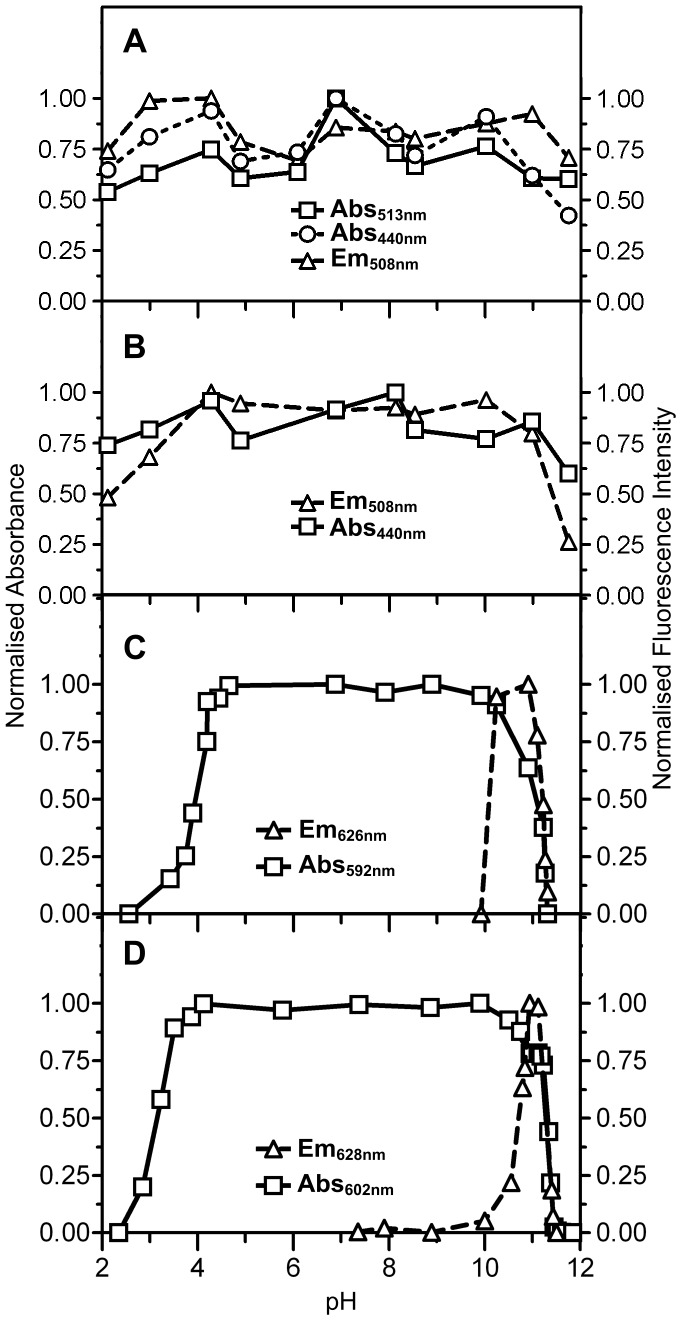
The effect of pH on the fluorescence emission and absorbance of Rtms5^Y67F^, Rtms5^Y67F/H146S^, Rtms5 and Rtms5^ H146S^. Absorbance and fluorescence emission spectra were determined at different pH in buffers of constant ionic strength for (A), Rtms5^Y67F^; (B), Rtms5^Y67F/H146S^; (C), Rtms5 and (D), Rtms5^ H146S^. Values shown are those at the 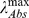 and 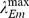 for each protein. Excitation was 440 nm for A and B, and 590 nm for C and D.

Finally, we investigated in further detail the effect of pH on the absorbance and fluorescence emission spectra of Rtms5^Y67F^ and Rtms5^Y67F/H146S^. The absorbance and fluorescence emission for both Rtms5^Y67F^ and Rtms5^Y67F/H146S^ remained remarkably stable over the range pH 3–11 (pK_a_ <3.0 absorbance and emission) (Fig. 5a and b). Changes in absorbance and emission observed outside this pH range (<3 and >11) are likely the result of nucleophilic attack on the acylimine linkage and loss of chromophore conjugation as already discussed (Fig. 3). In comparison absorbance by Rtms5 and Rtms5^H146S^ (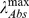, ∼ 592 nm) decreases significantly below pH ∼ 4 (pK_a_ 3.2 and 4.6 for Rtms5 and Rtms5^H146S^, respectively) (Fig. 5c and d) [17]. These proteins also show a significant increases in Φ_F_ at pH >10. The 4-hydroxybenzylidine moiety of the Rtms5 and Rtms5^H146S^ chromophores titrates between an anionic form (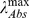, ∼ 592 nm) and neutral form (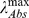, 450nm) [16], whereas the benzylidine moiety of the Rtms5^Y67F^ and Rtms5^Y67F/H146S^ chromophore, lacking a titratable group exists in a neutral form at all pH values (Fig. 2). Collectively these results indicate that the absorbance and fluorescence properties of Rtms5^Y67^ and in particular Rtms5^Y67F/H146S^ are stable over a wider range of pH compared to their tyrosine-containing counterparts, Rtms5 and Rtms5^H146S^.

### Structural Overview of Rtms5^Y67F^ and Rtms5^Y67F/H146S^

**Figure 6:**
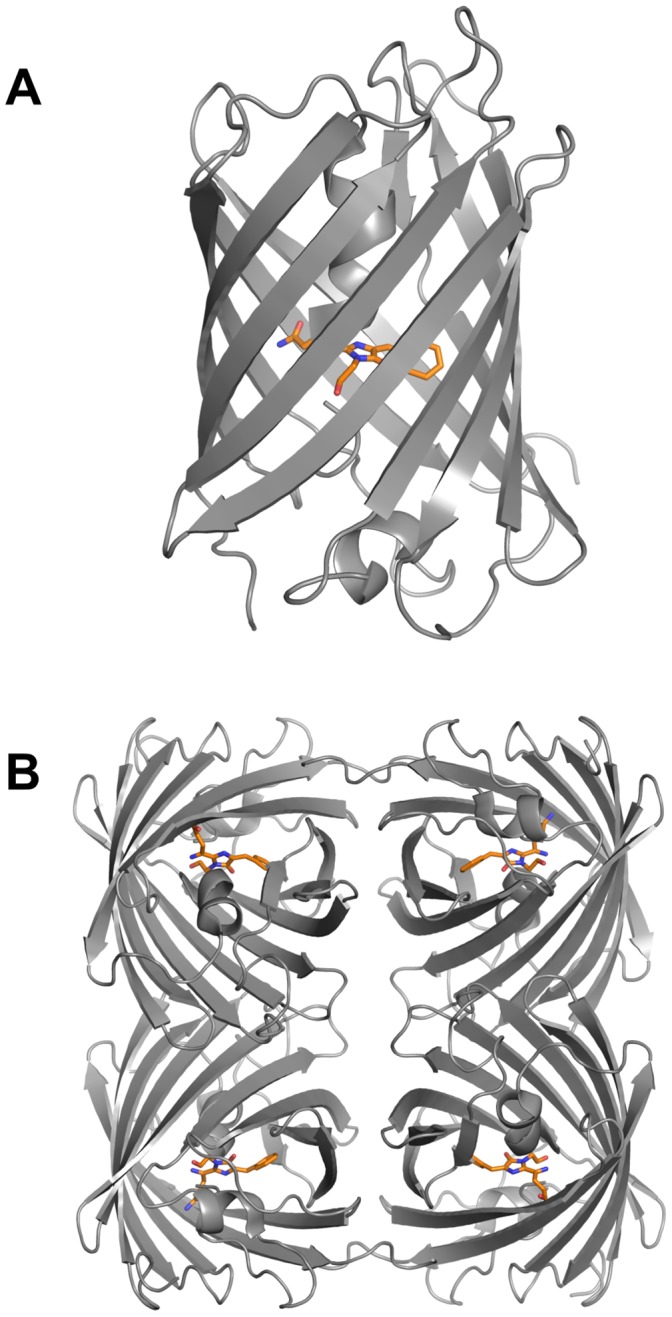
Rtms5^Y67F^ structure. A schematic ribbon representation of an isolated protomer (A) of Rtms5^Y67F^ showing the 11-stranded β-can motif typical of GFP-like proteins. The biological assembly as predicted by PISA is a 222 tetramer similar to Rtms5 (B). The chromophore is represented in stick format.

We have determined the X-ray crystal structure of Rtms5^Y67F^ and Rtms5^Y67F/H146S^. The crystallography and structural statistics are reported in Table 2. Each of the protomers in Rtms5^Y67F^ and Rtms5^Y67F/H146S^ consist of the same 11-stranded β-can motif (Fig. 6a) typical of members of the GFP-superfamily of proteins. Located at the core of the barrel is the circularised tri-peptide QFG chromophore maintaining covalent links to Cys65 and Ser69 of the main-chain. Within the asymmetric unit of Rtms5^Y67F^ there are 2 tetramers with 222 non-crystallographic symmetry (Fig. 6b) which both match the biological unit predicted by analysis using PISA [21] and the biological unit observed for Rtms5. Rtms5^Y67F/H146^ is also predicted to form a tetramer with222 non-crystallographic symmetry in the biological unit. The greatest rmsd value between protomer A and its 7 non-crystallographically symmetry related protomers of Rtms5^Y67F^ was 0.134 Å and, as such, the protomers are considered identical. Clear electron density for the Rtms5^Y67F^ chromophore was observed in each protomer with clear links to Cys65 and Ser69 while the density for the Rtms5^Y67F/H146S^ chromophore was more ambiguous.

**Table 2:** Rtms5^Y67F^ and Rtms5^Y67F/H146S^ data collection and refinement statistics.

Parameter	Rtms5^Y67F^	Rtms5^Y67F/H146S^
Beamline	APS IMCA-CAT	Australian Synchrotron MX-01
Resolution range (Å)	54.8-2.2(2.3-2.2)*	50.0-2.2(2.3-2.2)
Space group	C222_1_	P4_2_22
*a, b, c* (Å)	150.3, 186.1, 185.2	93.1, 93.1, 76.9
α, β, γ (°)	90.0, 90.0, 90.0	90.0, 90.0, 90.0
Total reflections	974,663	118,881
Unique reflections	130,962	17,639
Multiplicity	7.4(7.5)	6.7(6.6)
Mean I/σ(I)	7.1(1.6)	16.1(2.2)
Completeness (%)	100(100)	99(99)
R_merge_ ** (%)	8.9(45.6)	8.2(63.5)
*Refinement*		
Resolution Range (Å)	117.0-2.2(2.3-2.2)	36.6-2.2(2.3-2.2)
Completeness (%)	99.97(100)	100(100)
Reflections	124,333(9,125)	16,740(1,187)
R_factor_ (%)	15.42	19.68
R_free_ (%)	19.77	23.99
*Non-Hydrogen atoms*		
Protein	13,898	1,693
Chromophore	184	23
Water	1,416	144
I^−/^Cl^−^	30	6
*R.m.s. deviations*		
Bond lengths (Å)	0.024	0.022
Bond angles (°)	2.02	1.97
*Ramachandran plot*		
Most favored regions (%)	98.6	98.1
Allowed regions (%)	1.4	1.4
*B factors*		
Avg. main-chain (Å^2^)	26.87	52.38
Avg. side-chain (Å^2^)	29.65	54.14
Avg. water (Å^2^)	35.93	55.22
Avg. chromophore (Å^2^)	34.87	82.52

* Values in parentheses refer to the highest resolution shell.

** *R*_merge_ = Σ|Ihkl–<Ihkl>|/ΣIhkl.

### Chromophore Structure and Environment

In the following section we describe the chromophore structure and environment of Rtms5^Y67F^ and Rtms5^Y67F/H146S^ in relation to the parent protein Rtms5 [15]. Rtms5^Y67F^ and Rtms5^Y67F/H146S^ each contain a benzylidine imidazolinone chromophore derived from the tripeptide Gln-Phe-Gly (Fig. 2). In each variant the Gln66 Cα, originally in the sp^3^ hybrid conformation is planar and sp^2^ hybridised as observed for other Rtms5 structures [15], [16], [17]. This arrangement is consistent with the formation of an acylimine linkage extending the π-bonding system of the chromophore as suggested by the red-shifted spectral data (Fig. 3; Fig. 2).

**Figure 7:**
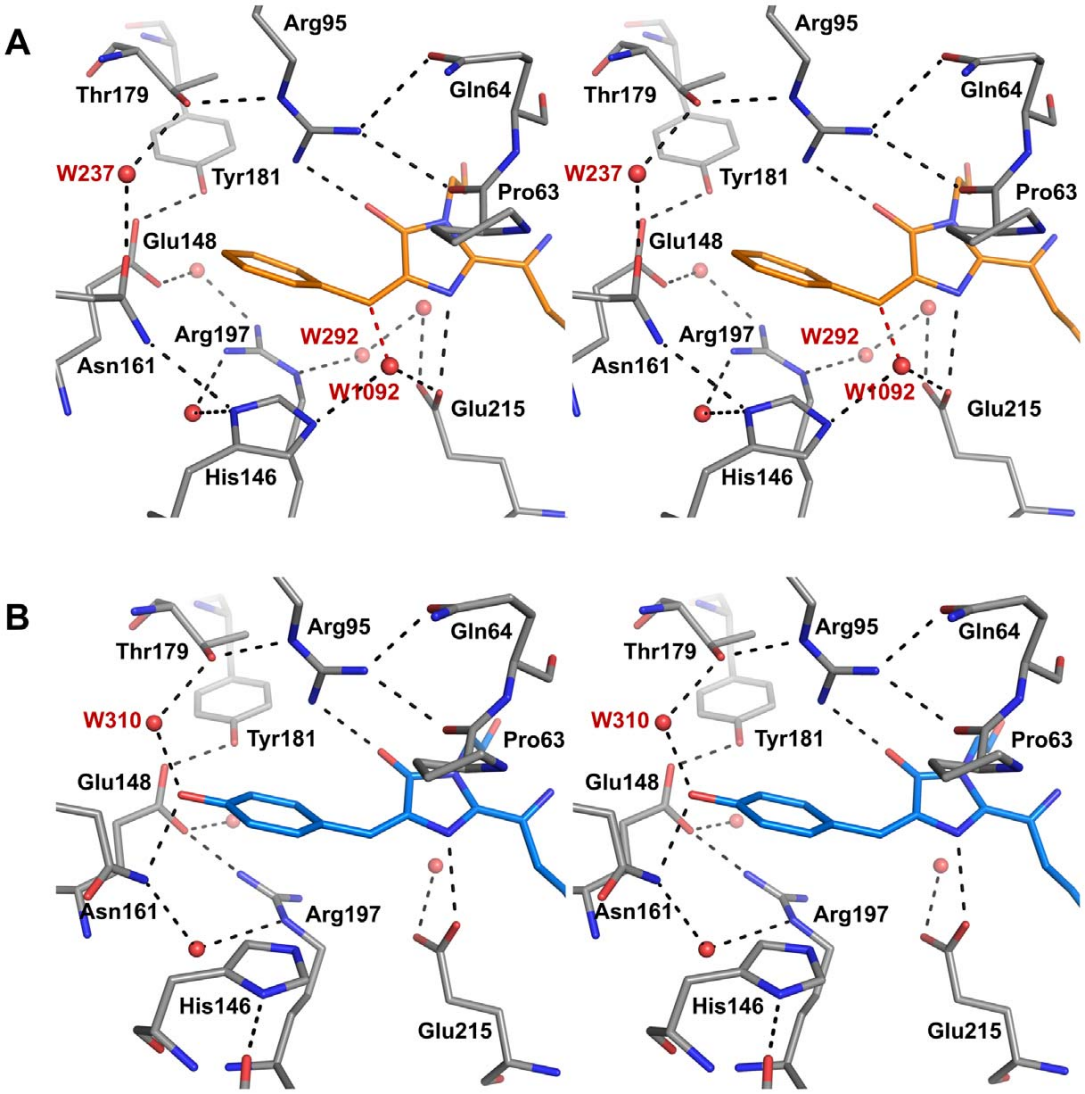
The chromophore environment of Rtms5^Y67F^ and Rtms5. Stereoviews are shown comparing the chromophore environments and H-bonding for Rtms5^Y67F^ (A) and Rtms5 (B). Chromophores are shown in orange (Rtms5^Y67F^) or blue (Rtms5). H-bonding is indicated by broken lines (corresponding distances are shown in Table 3). Waters are shown as red spheres. Two waters (W1092 and W2932) present in Rtms5^Y67F^ but not Rtms5, that contribute to differences in H-bonding are labelled. The distance between W1092 and Cβ2 of the methine bridge of the Rtms5^Y67F^ chromophore is 2.2 Å and highlighted by a red broken line. H-bonds between the 4-hydroxybenzylidine moiety of Rtms5 and Thr179 (water mediated) and Asn161 are not present in Rtms5^Y67F^.

Contacts between the Rtms5^Y67F^ chromophore (glutaminyl, imidazolinone and glycyl moieties) and the protein matrix are similar to those observed for Rtms5 (Table 3; Fig. 7; [15]). However, contacts between the respective protein matrix and the 4-hydroxybenzylidine of Rtms5 or benzylidine of Rtms5^Y67F^ are different. The 4-hydroxybenzylidine of Rtms5 is stabilised by a total of 17 van der Waals (vdw) interactions (Fig. 7b) [15] whereas the benzylidine moiety of Rtms5^Y67F^ is stabilised by only 14 vdw interactions. The vdw interactions in Rtms5^Y67F^ are contributed by His146, Arg197, Asn161, Glu148, Arg97 and Phe177 (Fig. 7a; Table 3).

**Table 3:** Rtms5^Y67F^ chromophore contacts.

Chromophore	Interacting protein atom(s) and distance (Å in parenthesis)*	Nature of interactions	
*Glutaminyl moiety*			
Oε1	Gln213Nε2 (3.3)	H-bond	
Nε1	Tyr14OH (3.1)	H-bond	
	Val44Cγ1 (3.4), Cβ (3.8), N (3.7), Thr43C (3.9), Cα (3.9), N (3.9),Gln42Cδ (3.7), Cγ (3.5), O (3.8), C (3.9)	vdw	
Cδ3	Val44Cγ1 (3.5)	vdw	
Cγ1	Cys65C (3.7), Gln42Cδ (3.9)	vdw	
Cβ1	Cys65C (3.4)	vdw	
Cα1	Cys65Cα (3.5)	vdw	
*Imidazolinone moiety*			
C1	Cys65C (3.6), Pro63C (3.8), Ser69N (3.1)		vdw
N2	Glu215Oε1 (2.9)		H-bond
	Glu215Oε2 (3.6), Cδ (3.6)		vdw
C2	Ile70Cδ1 (3.9)		vdw
	Arg95Nη1 (3.2), Ser69N (3.6)		vdw
O2	Arg95Nη1 (3.2), Nη2 (2.7)		H-bond
	Arg95Cζ (3.4), Ile70Cδ1 (3.3)		vdw
N3	Ser69N (2.7)		vdw
Cα2			
*Benzylidene moiety*			
Cβ2	Arg197Cδ (3.8)		vdw
Cγ2	Arg197Cδ (3.8), His146Cε1 (3.9)		vdw
Cδ1	His146Cε1 (3.3)		vdw
Cε1	Phe177Cδ1 (3.9), Asn161Cγ1 (3.6), His146Cε1 (3.8)		vdw
Cζ	Asn161Cγ (3.9), Glu148Cγ (4.3)		vdw
Cε2	Arg197Cζ (3.5), Glu148Cδ (3.8)		vdw
Cδ2	Arg197Cζ (3.9), Arg197Cδ (3.8)		vdw
*Glycyl moiety*			
Cα3	Trp93Cζ2 (3.7), Cys65C (3.8), Gln64C (3.8)		vdw
C	Cys65C (3.7)		vdw
O	Ser111Oγ (2.6 - HOH230 - 3.2), Gln64O (3.0 - HOH241 - 2.9)		Water-mediated H-bond

* Distances measured from within protomer A.

The 4-hydroxybenzylidine moiety of Rtms5 is stabilised by a water-mediated (W310) H-bond with Thr179 and an H-bond with Asn161 (Fig. 7b). However, in the absence of a hydroxyl group the benzylidine moiety of Rtms5^Y67F^ lacks such contacts. As a consequence the side-chain of Asn161 of Rtms5^Y67F^ is rotated around the Cα, and extends towards the 4-hydroxybenzylidine moiety of the Rtms5 chromophore, where Oδ2 maintains a water mediated H-bond with Oγ1 of Thr179, whilst Nδ2 forms an H-bond with Nδ1 of the imidazole ring of His146 (Fig 7a). Since the chromophores in both Rtms5^Y67F^ and Rtms5 are non-coplanar it can be concluded that contact with Thr179 does not contribute to stabilisation of this conformation.

Two waters (W292 and W1092) not observed in Rtms5 or Rtms5^Y67F/H146S^, contribute to differences in hydrogen bonding around the chromophore of Rtms5^Y67F^ (Fig. 7a). The Nε2 of His146 forms a water-mediated H-bond with Oε1 of Glu215 through water molecule W1092. Notably, this water is within 2.1 Å of Cβ2 of the chromophore methine bridge (Fig. 7a). It is possible that the proximity of W1092 to the methine bridge contributes to the observed red-shift in the absorbance spectrum of Rtms5^Y67F^ compared to that of Rtms5^Y67F/H146S^ (Fig. 1; Table 1) by coordinating increased electron pair density on the bridge of the chromophore [22]. A water-mediated H-bond is maintained between Oε2 of Glu148 and Nε of Arg197 through water A292. Additionally, the Glu215 carboxyl Oε1 H-bonds to N2 of the chromophore imidazolinone ring, while Glu215 Oε2 maintains water-mediated H bonds with Oγ Ser217 and Oγ1 Thr73 through water W292, and a water-mediated H-bond to N2 of the chromophore imidazolinone ring through water W247.

The imidazole ring of His146 in Rtms5^Y67F^ is rotated around Cβ towards the benzylidine ring and contributes to a significant increase in the non-coplanarity of the Rtms5^Y67F^ chromophore compared to the Rtms5 chromophore (Fig. 7a). The benzylidine moiety of Rtms5^Y67F^ is twisted out of plane with respect to the imidazolinone ring with tilt and twist angles of −178° and 53°, respectively averaged across all eight protomers (Table 4) whereas the 4-hydroxybenzylidine ring of Rtms5 is twisted out of plane with respect to the imidazolinone ring with tilt and twist angles of 170° and 43°, respectively [15].

**Table 4:** Measured angles for the chromophores of Rtms5 variants and selected fluorescent proteins.

Protein	Methine Bridge angle (°)*	Tilt (τ)	Twist (φ)
Rtms5^Y67F^	121 (±2)	-178 (±2)	53 (±3)
Rtms5^Y67F/H146S^	133	-178	43
Rtms5(1)	139	170	43
Rtms5^H146S(2)^	140	169	42
mCherry(3)	134	26	-13
mNeptune(4)	122	5	-9

* The measured angle between the Cα2-Cβ2 and Cβ2 and Cγ2 bonds of the chromophore. PDB files analysed in this table include.

(1) 1MOU,

(2) 1MOV,

(3) 2H5Q,

(4) 3IP2. Angles for Rtms5 are averaged across all 8 protomers and the SD shown.

The different constraints imposed by the protein matrix upon the Rtms5^Y67F^ and Rtms5 chromophores are reflected in the average angle for the Cα2-Cβ2-Cγ2 bond of the methine bridge (Fig. 2). The average angle of 121° for the Cα2-Cβ2-Cγ2 bond in Rtms5^Y67F^ is close to the ideal angle for this bond, compared to angles of 139° and 140° observed in Rtms5 and Rtms5^H146S^, respectively (Table 4).

**Figure 8:**
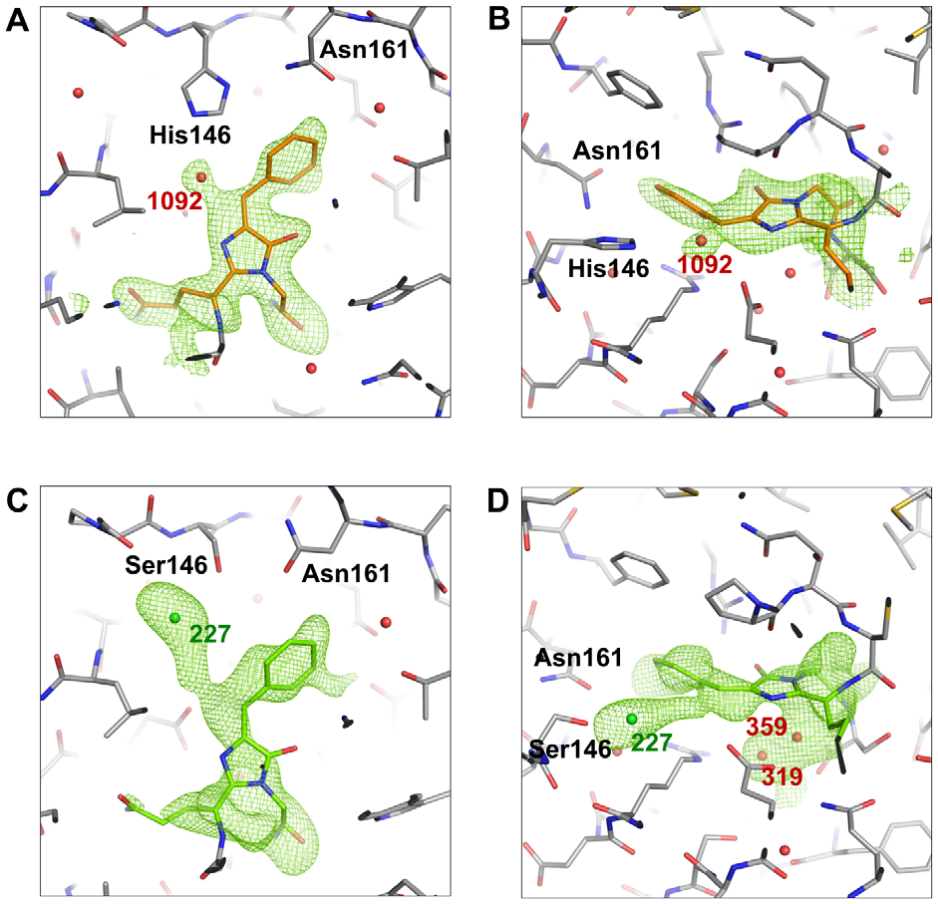
Simulated annealing omit maps for the chromophores of Rtms5^Y67F^ and Rtms5^Y67F/H146S^. Alternate views are shown for the non-coplanar chromophores of Rtms5^Y67F^ (A and B; orange) and Rtms5^Y67F/H146S^ (C and D; green). Nearby waters (numbered red spheres) were included in the omit map calculation. The omit map calculation for the Rtms5^Y67F/H146S^ chromophore included a nearby chloride (green sphere). The omit map indicates that the Rtms5^Y67F^ chromophore is in the *trans* conformation whilst the Rtms5^Y67F/H146S^ chromophore omit map is more ambiguous. The mesh representing the omit maps is contoured to 2.5σ. Difference maps showing the *trans* and *cis* Rtms5^Y67F/H146S^ chromophore conformations under different occupancies are shown in Fig. S2.

Compared to Rtms5^Y67F^, the structure of the Rtms5^Y67F/H146S^ chromophore is less well defined with B-factors higher than the side-chains of the surrounding residues. A simulated annealing omit map shows that compared to Rtms5^Y67F^, the Rtms5^Y67F/H146S^ chromophore is not well-defined in the electron density (Fig. 8). This effect may result from a reduced number of chromophore contacts as observed for Rtms5^Y67F^ when compared to Rtms5, together with the additional His146Ser substitution. Nevertheless, sufficient electron density exists to enable the modelling of a *trans,* non-coplanar Rtms5^Y67F/H146S^ chromophore. As a result of the His146Ser substitution, a pocket exists in Rtms5^Y67F/H146S^ with the potential to accommodate the chromophore in a *cis* conformation (Fig. S1). In order to investigate the possibility that the Rtms5^Y67F/H146S^ chromophore is mobile and is able to adopt alternate conformations, the *trans* and *cis* chromophore conformations were modelled at different occupancies. The difference maps showed increasing amounts of negative density in the position corresponding to the *cis* conformation as the occupancy of the *cis* chromophore approaches 1 (Fig. S2). The analysis suggests that the *trans* conformation of the Rtms5^Y67FH146S^ chromophore is favoured.

### Quantum Chemical Calculations

**Figure 9:**
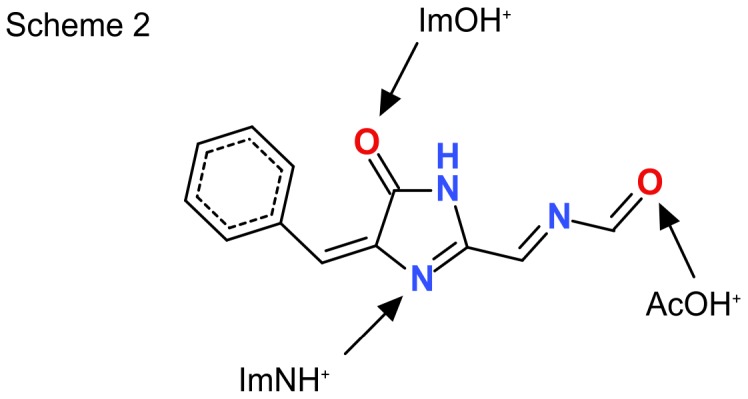
The chromophore model used for quantum chemical calculations. The chromophore model is truncated at a level consistent with earlier studies of acylimine-substituted FP chromophore models. The neutral unprotonated form is shown. The protonation sites for each of the three singly protonated forms are indicated.

In order to guide the assignment of the absorbance bands of the Rtms5^Y67F^ variants investigated in this study, we performed quantum chemical calculations of the electronic excitation energies of a truncated model of the chromophore. The chemical structure of the chromophore model is shown in Figure 9. The model is truncated at a level consistent with earlier studies of acylimine-substituted FP chromophore models, and includes all atoms that contribute to the π-electron system [23], [24]. We examined four distinct protonation states of the model: an unprotonated neutral form, and three singly protonated forms with the proton bound to the imidazolinone nitrogen site (ImNH^+^), the imidazolinone oxygen site (ImOH^+^), and the acylimine oxygen site (AcOH^+^). The excitation energies and dipole observables associated with the S_0_–S_1_ transition of the Rtms5^Y67F^ chromophore model are listed in Table 5.

The computational results were obtained for the truncated model in gas phase and any effects of the protein environment, both steric and electronic, are neglected. For this reason, the confidence that one can place on assignments based on these data is determined by the relative separation of the distinct absorbance bands in the proteins and the separation of excitation energies for different states of the model. Fortunately, the excitation energies of most of the states used in the calculations are quite distinguishable. However, we note that in all cases the optimized geometries of the models are planar. Non-planar distortions of the methine bridge are expected to provide a modest red-shift (on the order of 0.1 eV) [24]. Non-planarity of the acylimine linkage is expected to affect the absorbance to a smaller extent, because the conjugation through the imine nitrogen can occur even with significant twisting [23].

**Figure 10:**
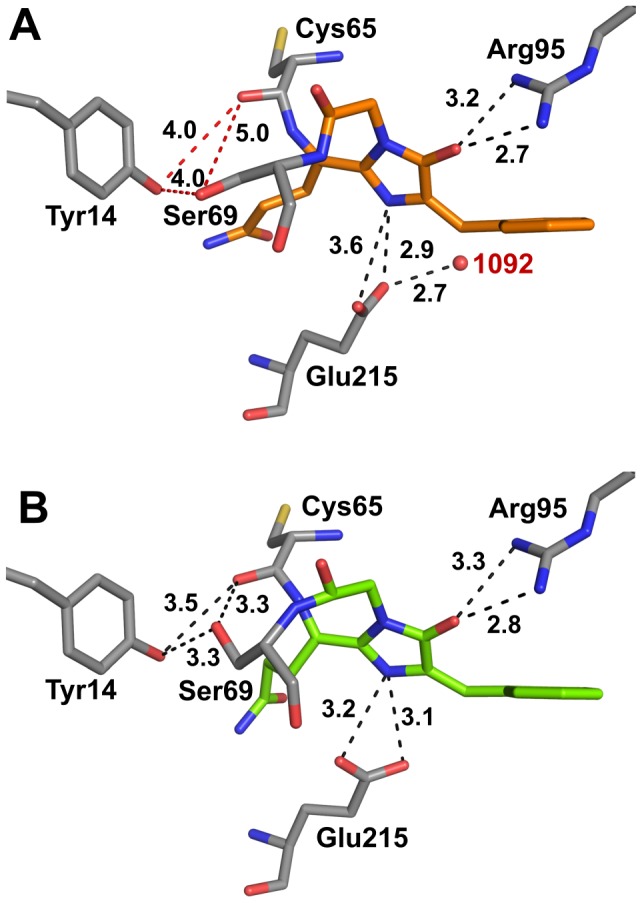
Chromophore contacts for Rtms5^Y67F^ and Rtms5^Y67F/H146S^. Selected contacts are shown for the chromophore of Rtms5^Y67F^ (A) and Rtms5^Y67F/H146S^ (B) highlighting the different positioning of the Ser69 side-chain relative to the acylimine oxygen. The charge associated with acylimine oxygen is thought to explain the existence of the 513 nm absorbing species in Rtms5^Y67F^.

Rtms5^Y67F^ but not Rtms5^Y67F/H146S^ has an absorbance band at 513 nm (Fig. 1). The calculated excitation energy of the state protonated at the acylimine oxygen (AcOH^+^) is significantly redder than the neutral chromophore (368nm) (Table 5). This suggests that the absorbance band near 513 nm, characteristic of Rtms5^Y67F^ should not be attributed to an unprotonated chromophore species. Instead, this band is more reasonably assigned to a species that is protonated at the acylimine oxygen. A difference in the position of the side-chain of Ser69 in Rtms5^Y67F^ compared to Rtms5^Y67F/H146S^ lends support to this idea. The Oγ of Ser 69 and ηOH of Tyr 14 in Rtms5^Y67F/H146S^ are within H-bonding distance of the acylimine oxygen (Fig. 10). Rotation of the Ser69 side-chain and repositioning of the acylimine oxygen in Rtms5^Y67F^ place them beyond hydrogen bonding distance suggesting a change in the charge associated with the acylimine oxygen.

**Table 5:** Results of quantum chemistry calculations† on neutral and singly protonated forms of the Rtms5^Y67F^ chromophore.

Protonation Site	Relative S_0_ Energy (kcal/mol)	S_0_–S_1_ Excitation Energy (eV)	S_0_–S_1_ TransitionDipole Norm (*e*Å)	S_0_–S_1_ DifferenceDipole Norm (*e*Å)	S_0_–S_1_ Trans./Diff.Dipole Angle
		*Wavelength (nm)*			
None	N/A††	3.37	1.5	1.4	5^0^
		*368*			
ImNH^+^	0.0	2.62	1.8	2.2	4^0^
		*473*			
ImOH^+^	9.4	2.95	1.9	0.4	2^0^
		*420*			
AcOH^+^	9.6	2.28	2.5	0.5	31^0^
		*545*			

† Results of SA2-CAS(4,3)*MS-MRPT2//cc-pvdz calculations at MP2//cc-pvdz optimized geometries for models of the *trans* isomer.

†† Relative energies of species with different constituency cannot be compared; accordingly, only ground state energies of singly protonated species are reported.

## Discussion

This is the first report describing an FP with green fluorescence emission (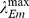, 500–520 nm) that does not have tyrosine as the aromatic amino acid in the chromophore tripeptide. Only two other FPs, the cyan emitting mBlueberry 2 (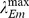, 467 nm) and mBlueberry 1 [25], are presumed to contain the same chromophore structure as Rtms5^Y67F^ and Rtms5^Y67F/H146S^. mBlueberry 2 was derived from the acylimine-containing red fluorescent mCherry by introduction of number of amino acid substitutions including a Tyr to Phe substitution at position 67. In the absence of a an X-ray crystal structure for mBlueberry 2 the reasons for the marked difference in emission maxima (∼ 40 nm) between mBlueberry 2 and the Rtms5^Y67F^ variants (Table 2) are unclear but presumably arise from altered contacts of the chromophore with the surrounding amino acid side-chains. It is known that subtle changes in chromophore contacts can generate significant differences in the emission spectra. For example, the position of the positively charged side-chain of Arg197 relative to the 4-hydroxy benzylidine moiety is, in part, believed to be responsible for producing the significantly red-shifted spectra of mNeptune (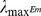, 655 nm) [14]. In Rtms5^Y67F^ the same side-chain of Arg197 is within vdw distance of the benzylidine ring (Table 3; Fig. 7a; Fig. S1) whereas in mBlueberry1 and mBlueberry2 the charged side chain of Arg197 is substituted by the non charged side-chain of isoleucine [25], a change that would be consistent with the blue-shifted spectra observed for the mBlueberry variants.

The weak fluorescence emission observed for both Rtms5 and Rtms5^H146S^ (Φ_F,_ 0.004 and 0.02, respectively) has been attributed previously to their *trans* non-coplanar chromophores [15]. A significant increase in fluorescence emission (20-fold; Φ_F,_ 0.16) observed for Rtms5^H146S^ at alkaline pH (see Fig. 5d) was accompanied by an increased proportion of a *cis*-coplanar chromophore as observed in the X-ray crystal structure [16]. Since Rtms5^Y67F^ and Rtms5^Y67F/H146S^ are considerably more fluorescent (Φ_F,_ 0.11 and 0.75, respectively) compared to their Rtms5 parents, we were surprised by the lack of evidence for a *cis*-coplanar chromophore in their structures. The poor electron density corresponding to the chromophore in Rtms5^Y67F/H146S^ suggests it is mobile, and may adopt alternate conformations. However, the difference maps for *trans* and *cis* Rtms5^Y67F/H146S^ chromophore conformations under different occupancies indicated that the *trans* conformation is favoured (Fig. S2) leaving no clear explanation for the increased Φ_F_ of these proteins.

The chromophores in each of the bright red fluorescent EqFP611 and TagRFP [26], [27] are *trans*-coplanar suggesting that in different proteins a *cis* or *trans* chromophore can be highly fluorescent, providing they can adopt a coplanar conformation. It is possible that the fluorescent chromophore species in Rtms5^Y67F/H146S^ is *trans*-coplanar. Our data for Rtms5^Y67F/H146S^ show that contact between the benzylidine moiety of the chromophore and the side-chain of Arg197 prevents a coplanar conformation (Fig. S1). We attempted to model an alternative orientation of the Arg197 side-chain that allows a coplanar chromophore (Fig. S3). In this model the distance between the side-chain of Arg197 and the benzylidine ring has increased providing the room to accommodate a coplanar chromophore (Fig. S3c and d). In the case of the non-coplanar chromophore stabilisation of the Arg197 side-chain is provided by contact with the side chain of Glu148 and H-bonds mediated by W329 and W319 (Fig. S3a and b), whereas in the case of the coplanar chromophore, stabilisation is provided by H-bonds mediated through W329, W319 and W282 (Fig. S3c and d). How and when movement of the Arg197 side-chain takes place in Rtms5^Y67F/H146S^ is not clear. However, repositioning of the side-chain of a histidine at the same amino acid location is a key feature of the molecular mechanism for photoswitching of DronPA [28]. On illumination with excitation light repositioning of His197 in DronPA promotes isomerisation of the chromophore from a *trans* non-coplanar, non fluorescent form to a *cis* coplanar, brightly fluorescent form. If our model is correct Rtms5^Y67F/H146S^ and Rtms5 belong to very small group of FPs that have a fluorescent *trans* chromophore conformation. Further studies of Rtms5^Y67F/H146S^ are required to investigate the validity of this model.

We were intrigued as to the source of the 513 nm species in Rtms5^Y67F^. The results of chemical quantum calculations suggest this species may arise from a protonation event involving the acylimine oxygen (AcOH^+^; Scheme 2). This idea is given additional support by the structural data that suggests a change in the position of the side-chain of Ser 69 in Rtms5^Y67F^ compared to Rtms5^Y67F/H146S^ (Fig. 9). Interaction of the acylimine oxygen with the protein matrix appears to be important for generating a red-shift in the spectra of other FPs [29]. A hydrogen bond between the side-chain of Glu16 and the acylimine carbonyl has been suggested to be important for generating the red-shifted optical spectra of the far-red fluorescent mPlum [13].

The data in Table 5 also suggest that the absorbance band near 440 nm (Fig. 1), characteristic of both Rtms5^Y67F^ and Rtms5^Y67F/H146S^ should not be attributed to an unprotonated chromophore species. Instead, this band is more reasonably assigned to a species that is protonated at either the nitrogen (ImNH^+^) or oxygen site (ImOH^+^) on the imidazolinone ring. Although the excitation energy calculated for the ImOH^+^ model is closer to the experimentally measured energy gap, the weight of precedent favors assignment to a nitrogen-protonated ImNH^+^ species. Protonation at either of these two positions might be expected be reflected in altered chromophore/protein matrix contacts. Although differences exist in the H-bond network around the chromophore in the Rtms^Y67F^ variants compared to their tyrosyl-containing counterparts (Rtms5 and Rtms5^H146S^) (Fig. 7) in each case an H-bond exists between N2 and O2 of the imidazalinone ring and the Glu215 carboxyl and Arg95, respectively (Fig. 7). We have previously reported that the Rtms5 chromophore is not protonated [16], [30].

There are few reports in the literature of FPs containing a phenylalanine in position 67 of the chromophore. The phenylalanine substituted variants reported here represent an alternative platform on which to develop fluorescent proteins with green emissions (500–520 nm) and superior pH stability. These proteins may also have a fluorescent *trans* chromophore conformation. Rtms5^Y67F^ and Rtms5^Y67F/H146S^ have the most pH-stable (pK_a_, 3.5) green emissions of any FPs (500–520 nm) reported to date. The green emissions of Sapphire FPs having been reported previously to be the most stable to pH (pK_a_ 4.9) [31]. This feature of Rtms5^Y67F^ and Rtms5^Y67F H146S^ can be attributed to the benzylidine moiety which lacks a titratable group. The chromophore of the pH-stable blue-emitting Sirius contains the same benzylidine moiety, and has a pK_a_ <3.0 [8]. The Sapphire chromophore contains a 4-hydroxy benzylidine moiety [31]. The ability of Rtms5^Y67F^ and Rtms5^Y67F/H146S^ to fluoresce with little attenuation down to ∼ pH 3.5, may prove useful for engineering new improved biosensors for monitoring autophagy in live cells [32]. Autophagy is an important cellular process characterised by the delivery of material to the acidic (pH 4.8) lumen of the lysosome for degradation.

Rtms5^Y67F^ and Rtms5^Y67F/H146S^ are obligate tetramers. However, we recently described a monomer of Rtms5 called Ultramarine [33] that represents a starting point to develop monomer forms of the phenylalanine-substituted FPs, thereby allowing them to be used as fusion partners with other proteins of interest.

## Materials and Methods

### Mutagenesis, Protein Expression and Purification

Expression vectors encoding Rtms5^Y67F^ or Rtms5^Y67F/H146S^ were constructed by site-directed mutagenesis (QuickChange, Invitrogen) using the primer pair 5′-caccacagtgtcagttcggaagcataccattc-3′ and 5′-gaatggtatgcttccgaactgacactgtggtg-3′ and expression vectors pQE10:Rtms5 or pQE10:Rtms5^H146S^ as templates.

Rtms5^Y67F^ and Rtms5^Y67F/H146S^ proteins were expressed in the NovaBlue (λDE3) strain of *E. coli* (Novagen) and purified by Ni-NTA chromatography as described [15]. For crystallization purposes proteins were subjected to chromatography on a S200 size exclusion column equilibrated in 20 mM Tris-HCl, pH 8.0, 300 mM NaCl. Fractions containing Rtms5^Y67F^ or Rtms5^Y67F/H146S^ tetramer were pooled and concentrated to 15 mg.ml^−1^ ready for crystallization trials by the hanging drop vapour diffusion technique.

### Spectrometry

Fluorescence spectra were determined using a Varian Eclipse fluorescence spectrophotometer (Melbourne, Australia). Φ_F_ values were determined for proteins (in 20 mM Tris–HCl (pH 8.0), 300 mM NaCl) at 25°C as described [15], [34] using solutions of Rhodamine 101 (Φ_F_, 1.0) in buffer as standard. Absorbance spectra were determined using a Varian Cary 50 spectrophotometer. For pH titrations, proteins in 20 mM Tris-HCl (pH 8.0) were diluted (∼100-fold) as required into selected 0.1 M buffers [16], [17]. Absorbance spectra were recorded at 24°C after 30 sec gentle mixing. Sample pH was monitored using a micro-pH probe. Data from a single a determination are presented.

### Crystallization and Structural Determination

Crystals of Rtms5^Y67F^ and Rtms5^Y67F/H146S^ that appeared brown or pale green, respectively were obtained at 20°C via the hanging drop method. Protein (15 mg.ml^−1^) in 20 mM Tris, 300 mM NaCl, pH 8.0 was mixed 1∶1 or 1∶2 with crystallization solutions, respectively. Numerous small crystals were obtained using conditions reported for Rtms5 [15], [16], [17]. Further optimisation of the crystallisation conditions led to diffraction quality crystals. Rtms5^Y67F^ crystals (0.1–0.2 mm in length) were obtained using a crystallization solution composed of 22% PEG 3350 and 0.34 M KI buffered with 0.2 M Tris-HCl pH 8.5 in 3 µl hanging drops (1∶2 protein/crystallization solution ratio). Rtms5^Y67H/H146S^ crystals 0.1–0.2 mm in length were obtained using a crystallization solution with 21% PEG 3350, 0.36 M KI, and 25% glycerol buffered with 0.2 M Tris pH 8.5 in 3 µl hanging drops (1∶2 protein/crystallization solution ratio). Rtms5^Y67F^ crystals were flash frozen prior to data collection using 30% (v/v) glycerol in the precipitant as cryoprotectant. Crystals were transferred stepwise (5% increments) into increasing amounts of glycerol over a time period of 2 h. Rtms5^Y67F/H146S^ crystals were dipped in perfluoropolyether oil (PFO-X175/08, Hampton Research) for 1 min before vitrification in a nitrogen-gas stream maintained at 100 K.

Diffraction images for Rtms5^Y67F^ and Rtms5^Y67H/H146S^ were collected at the APS IMCA-CAT beamline in Chicago (USA), and at the MX-1 beamline of the Australian Synchrotron, respectively.

Data integration was carried out with the HKL software package (http://www.hkl-xray.com) [35] for Rtms5^Y67F^ and with XDS (http://xds.mpimf-heidelberg.mpg.de) [36] for Rtms5^Y67F/H146S^. Molecular replacement for both Rtms5^Y67F^ and Rtms5^Y67F/H146S^ was carried out with Phaser [37] included in the CCP4 program suite (http://www.ccp4.ac.uk/ccp4i_main.php) [38]. The initial search probe used in Phaser was the wild-type Rtms5 model (1MOV) trimmed of water molecules, residue 146, and the chromophore. The top-scoring Phaser solution for Rtms5^Y67F^ consists of 8 protomers arranged into a pair of 222 tetramers in its asymmetric unit while the top-scoring solution for Rtms5^Y67F/H146S^ had a single protomer in the asymmetric unit.

Restrained refinement of Rtms5^Y67F^ and Rtms5^Y67F/H146S^ models was carried out in Refmac5 [39] with automatic weighting interspersed with rounds of model building in WinCoot (http://www.ysbl.york.ac.uk/~lohkamp/coot/wincoot.html) [40]. TLS refinement was used in the last few rounds of refinement in Refmac5. Models were checked with Molprobity (http://molprobity.biochem.duke.edu) [41] to guide model building. Tight main-chain and medium side-chain NCS restraints were applied in Refmac5 to residues 8–223 in early rounds of refinement of Rtms5^Y67F^ which was relaxed in later rounds of refinement. Rounds of simulated annealing refinement in Phenix (http://www.phenix-online.org) [42] were used to reduce bias and calculate difference omit maps to guide model building. Water molecules were placed into peaks in the *F_O-_F_C_* map and kept in the model if they were located within hydrogen-bonding distance of chemically reasonable groups, visible at 3.0σ map contour level, and possessed a B-factor <80 Å^2^. Strong peaks observed in the *F_O-_F_C_* map too large to be waters were modelled as chloride ions while even stronger peaks were modelled as iodide ions (both included in the crystallization conditions).

The monomer library definitions and PDB coordinates of the QFG chromophore were created using the CCP4 Monomer Library Sketcher [43] by inputting then editing the coordinates of the wild-type CRQ chromophore. The QFG chromophores were then placed into the electron density using WinCoot. A chloride ion was placed into density near the Rtms5^Y67F/H146S^ chromophore proximal to Ser146 in a position shown to be accessible to halides in Rtms5^H146S^ [17].

Validation of the final Rtms5^Y67F^ and Rtms5^Y67F/H146S^ models prior to deposition through PDBj ADIT was carried out using Molprobity along with SFcheck [44], Procheck [45], and Rampage [46] from the CCP4 software suite. The final Rtms5^Y67F^ model was refined to R_factor_ 15.42% and R_free_ 19.77% with 98.6% and 1.4% of residues in the favored and allowed regions of the Ramachandran plot, respectively, with none in the generously or disallowed regions. The final Rtms5^Y67F/H146S^ model was refined to R_factor_ 19.68% and R_free_ 23.99% with 98.1% and 1.4% of residues in the favored and allowed regions of the Ramachandran plot, respectively, with none in the generously or disallowed regions. The coordinates and structure factors for Rtms5^Y67F^ and Rtms5^Y67F/H146S^ have been deposited in the Protein Data Bank (3VIC and 3VK1, respectively). Biological assemblies for Rtms5^Y67F^ and Rtms5^Y67F/H146S^ were predicted using PISA included in the CCP4 program suite [21]. A summary of data collection and refinement statistics is presented in Table 2.

### Quantum Chemical Calculations

For each protonation state examined, we optimized the geometry of the model using Møller-Plessett 2^nd^ order perturbation theory [47] and a cc-pvdz basis set [48] (MP2//cc-pvdz). At these geometries, we calculated the excitation energies, transition dipole and difference dipole moments using multi-state multi-reference 2^nd^ order perturbation theory [49], [50] on a four-electron, three-orbital two-state averaged complete active space self-consistent field wavefunction, again with a cc-pvdz basis set [48] (SA2-CAS(4,3)*MS-MRPT2//cc-pvdz). This protocol has previously been used to study the halochromism of GFP chromophore models [51], [52], [53]. All calculations were carried out using the MOLPRO software package (http://www.molpro.net) [54].

## Supporting Information

Figure S1 **The chromophore cavities of Rtms5^Y67F^ and Rtms5^Y67F/H146S^.** Orthogonal cutaway views are shown for Rtms5^Y67F^ (A and B) and Rtms5^Y67F/H146S^ (C and D). The side-chain of His146 stabilises the *trans* conformation of the Rtms5^Y67F^ chromophore. The His146Ser substitution (C) creates a pocket with the potential to accommodate an Rtms5^Y67F/H146S^ chromophore with a *cis* conformation. The non-coplanar conformation of the chromophores in both Rtms5^Y67F^ and Rtms5^Y67F/H146S^ is stabilised by the side-chains of Arg96 and Arg197 (C and D). Waters are shown as red spheres.
(TIF)

Figure S2 **Difference maps showing the** ***trans*** **and** ***cis*** **Rtms5^Y67F/H146S^ chromophore conformations under different occupancies.** Occupancy ratios (*trans/cis*) are 0.0/1.0, (A); 0.25/0.75, (B); 0.5/0.5, (C); 0.75/0.25, (D) and 1.0/0.0 (E). The positive (green mesh) and negative (red mesh) difference maps are contoured to +2.5σ and −2.5σ, respectively. The *trans* chromophore conformation is favoured in Rtms5^Y67F/H146S^. A nearby chloride ion (green sphere) was omitted from the map calculation.
(TIF)

Figure S3 **A model showing the chromophore cavity of Rtms5^Y67F/H146S^ with a hypothetical** ***trans*** **-coplanar chromophore.** Orthogonal views of the *trans* Rtms5^Y67F/H146S^ chromophore in a *trans* non-coplanar as suggested by the X-ray structure (A and B), and modelled in a *trans* coplanar conformation (C and D) are shown. The conformation of the Arg197 residue, which contacts the benzylidene moiety of the chromophore (pink dashed lines, distances in Å numbered in pink) restricts the possibility of a *trans* coplanar chromophore (A). The conformation of Arg197 is stabilised by H-bonds (black dashed lines, distances in Å shown numbered in black) to two nearby water molecules (red spheres, numbered in red) and to Glu148 (B). Repositioning of the Arg197 side chain (C) creates a space in which a *trans* coplanar chromophore could be accomodated. The side-chain of Arg197 in is stabilised by different contacts (D). A nearby chloride is shown (green sphere). The hypothetical model was created in WinCoot, avoiding major clashes with nearby atoms, and only the rearrangment of the Arg197 side chain has been considered.
(TIF)

Figure S4 **Hypothetical resonance structures for the chromophore model.**
(TIF)

Text S1 (DOCX)

Scheme S1 (TIF)

Scheme S2 (TIF)
