# Supplementary figures and images for: A Green Fluorescent Protein Containing a QFG Tri-Peptide Chromophore: Optical Properties and X-Ray Crystal Structure
Source: PLoS One. 2012 Oct 10;7(10):e47331. doi: 10.1371/journal.pone.0047331 (PMC3468514; doi:10.1371/journal.pone.0047331)

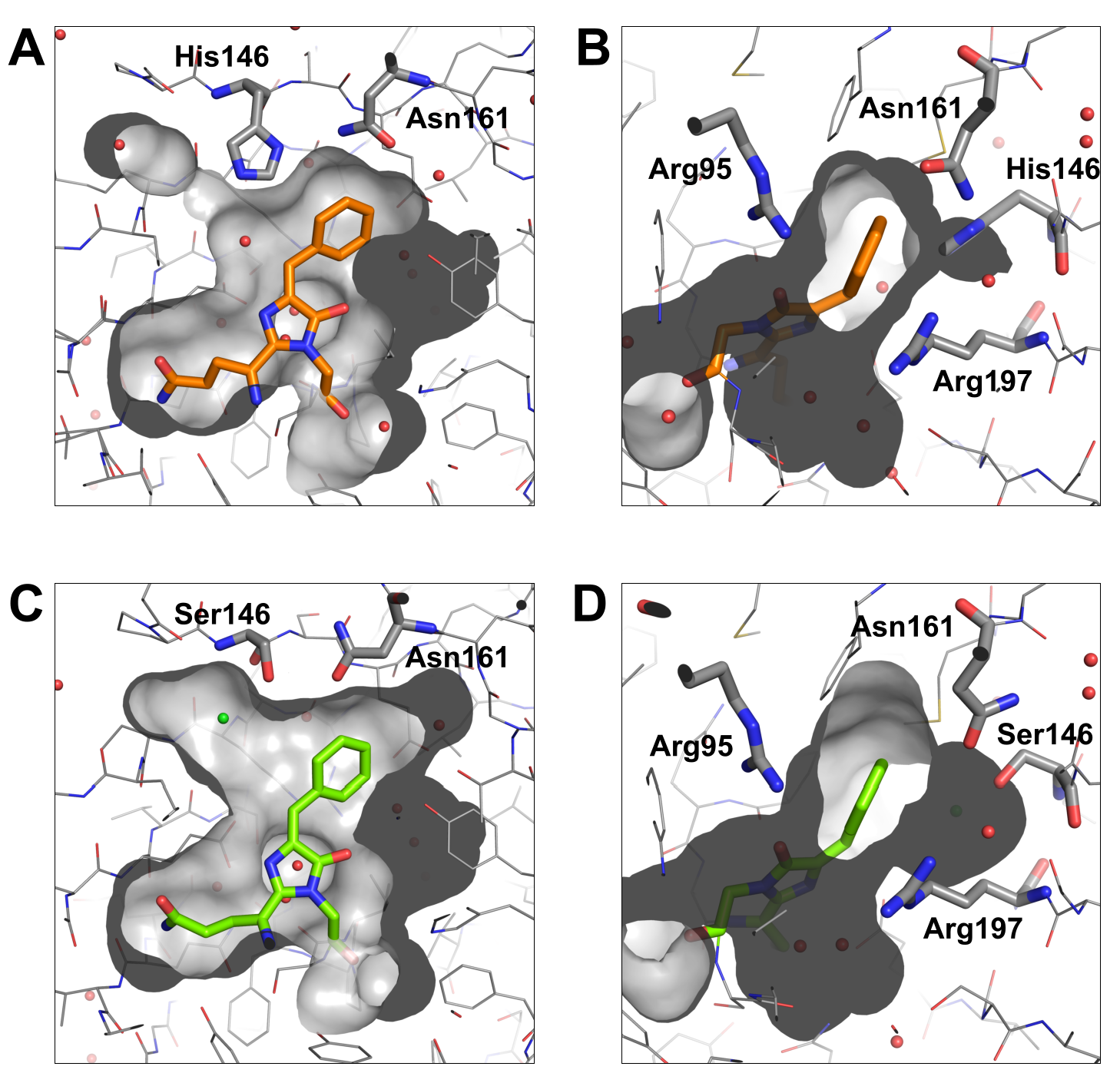

Supplement: Figure S1 — The chromophore cavities of Rtms5Y67F and Rtms5Y67F/H146S. Orthogonal cutaway views are shown for Rtms5Y67F (A and B) and Rtms5Y67F/H146S (C and D). The side-chain of His146 stabilises the trans conformation of the Rtms5Y67F chromophore. The His146Ser substitution (C) creates a pocket with the potential to accommodate an Rtms5Y67F/H146S chromophore with a cis conformation. The non-coplanar conformation of the chromophores in both Rtms5Y67F and Rtms5Y67F/H146S is stabilised by the side-chains of Arg96 and Arg197 (C and D). Waters are shown as red spheres. (TIF) [file pone.0047331.s001.tif]

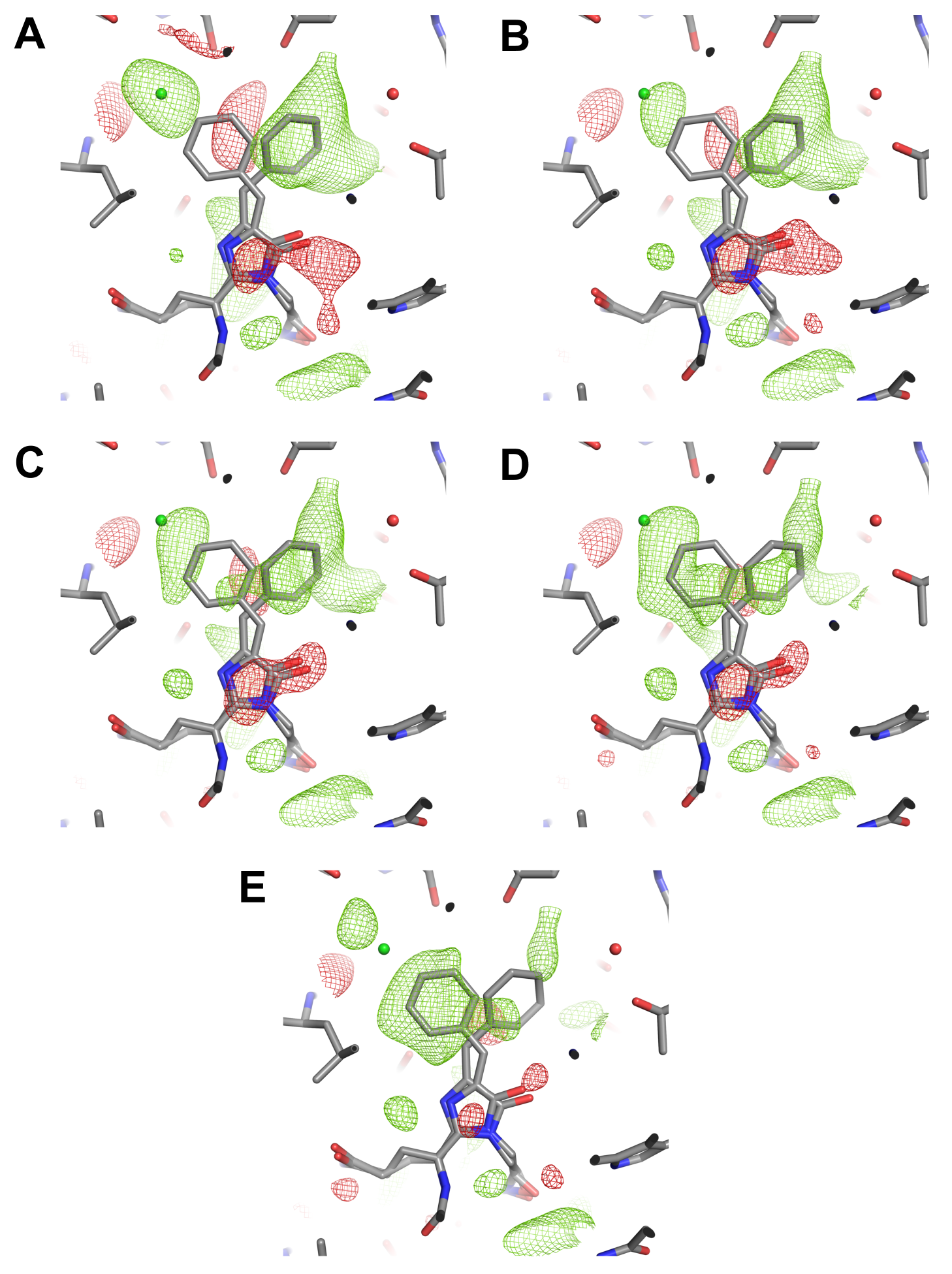

Supplement: Figure S2 — Difference maps showing the trans and cis Rtms5Y67F/H146S chromophore conformations under different occupancies. Occupancy ratios (trans/cis) are 0.0/1.0, (A); 0.25/0.75, (B); 0.5/0.5, (C); 0.75/0.25, (D) and 1.0/0.0 (E). The positive (green mesh) and negative (red mesh) difference maps are contoured to +2.5σ and −2.5σ, respectively. The trans chromophore conformation is favoured in Rtms5Y67F/H146S. A nearby chloride ion (green sphere) was omitted from the map calculation. (TIF) [file pone.0047331.s002.tif]

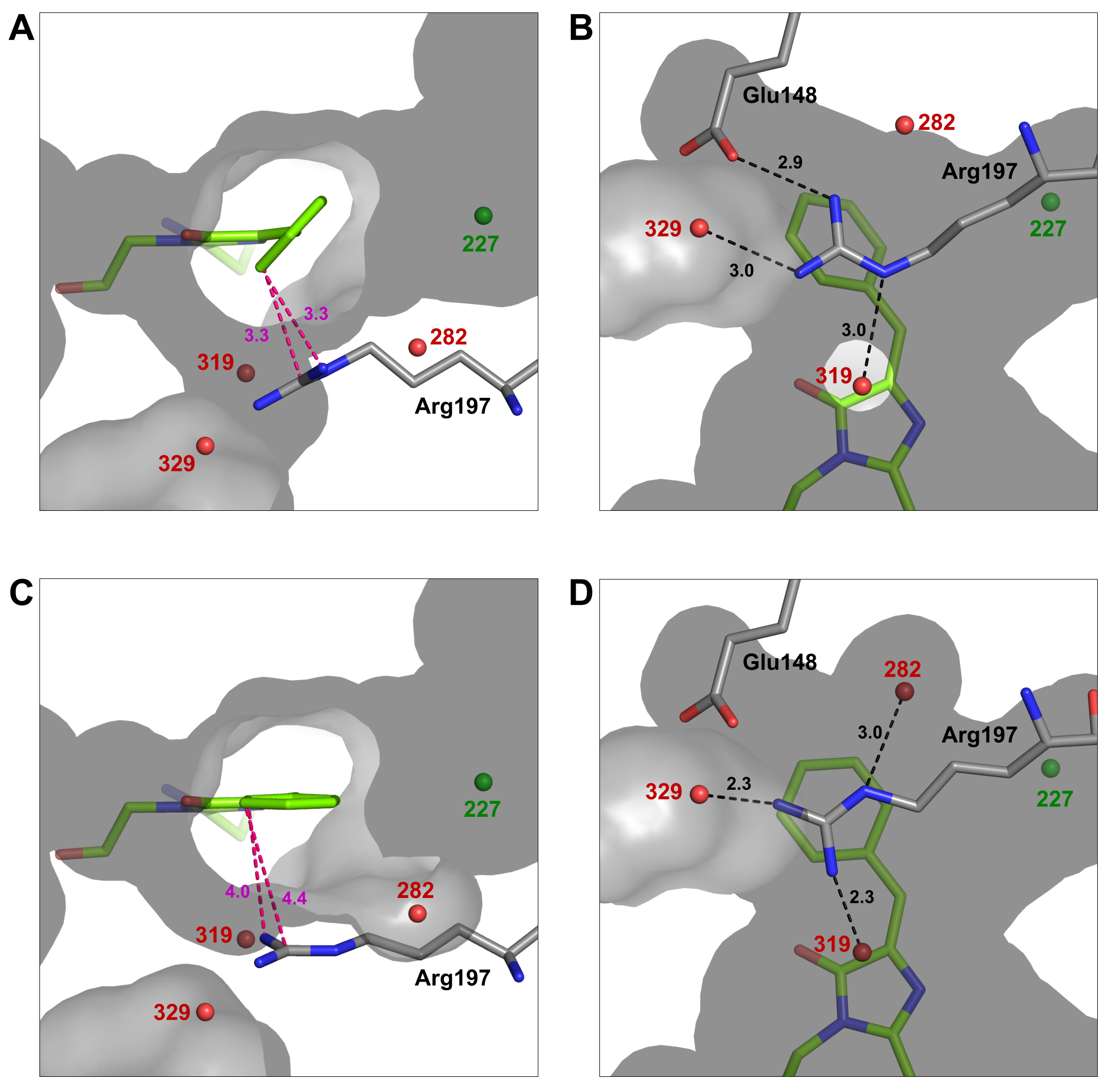

Supplement: Figure S3 — A model showing the chromophore cavity of Rtms5Y67F/H146S with a hypothetical trans -coplanar chromophore. Orthogonal views of the trans Rtms5Y67F/H146S chromophore in a trans non-coplanar as suggested by the X-ray structure (A and B), and modelled in a trans coplanar conformation (C and D) are shown. The conformation of the Arg197 residue, which contacts the benzylidene moiety of the chromophore (pink dashed lines, distances in Å numbered in pink) restricts the possibility of a trans coplanar chromophore (A). The conformation of Arg197 is stabilised by H-bonds (black dashed lines, distances in Å shown numbered in black) to two nearby water molecules (red spheres, numbered in red) and to Glu148 (B). Repositioning of the Arg197 side chain (C) creates a space in which a trans coplanar chromophore could be accomodated. The side-chain of Arg197 in is stabilised by different contacts (D). A nearby chloride is shown (green sphere). The hypothetical model was created in WinCoot, avoiding major clashes with nearby atoms, and only the rearrangment of the Arg197 side chain has been considered. (TIF) [file pone.0047331.s003.tif]

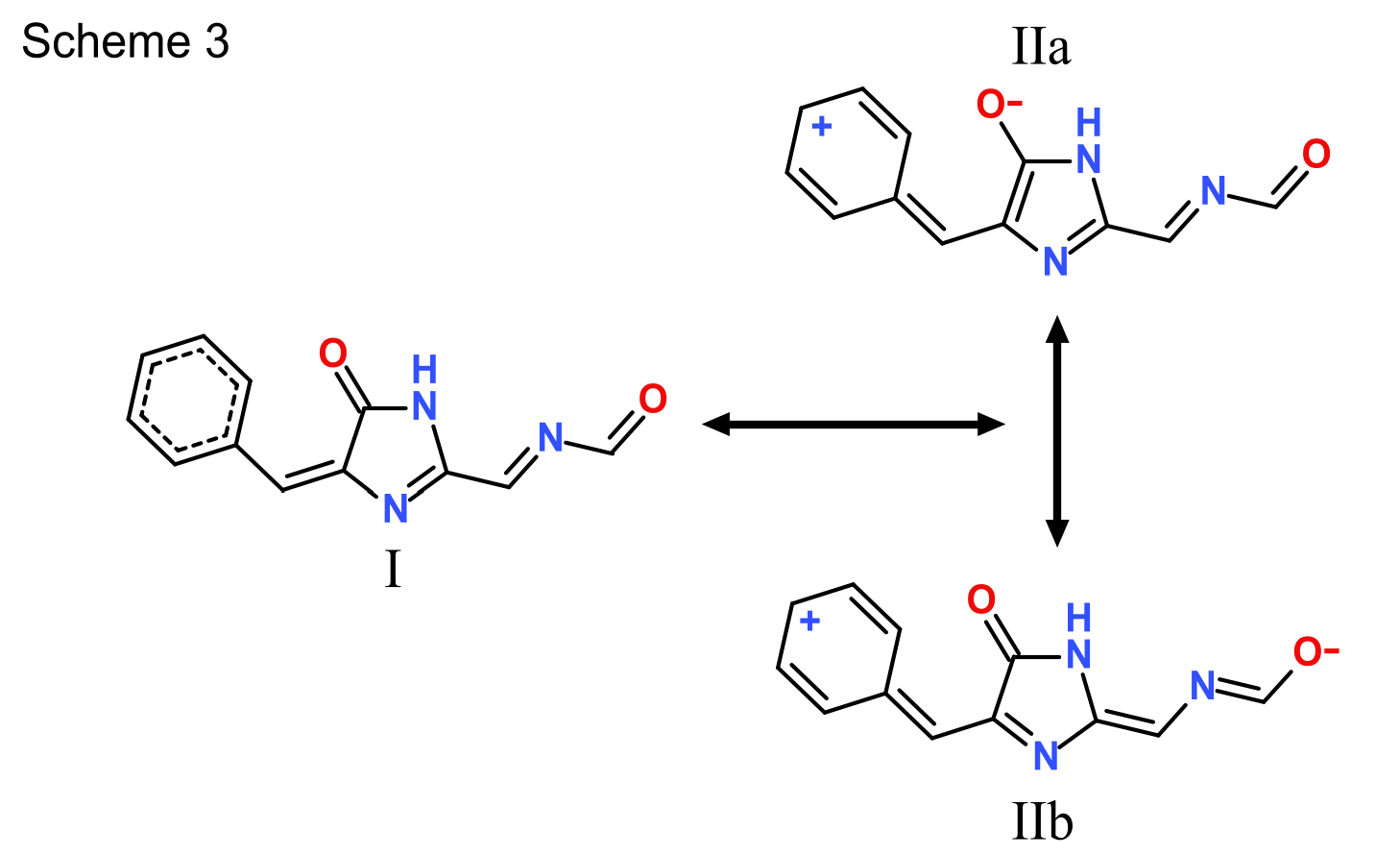

Supplement: Figure S4 — Hypothetical resonance structures for the chromophore model. (TIF) [file pone.0047331.s004.tif]

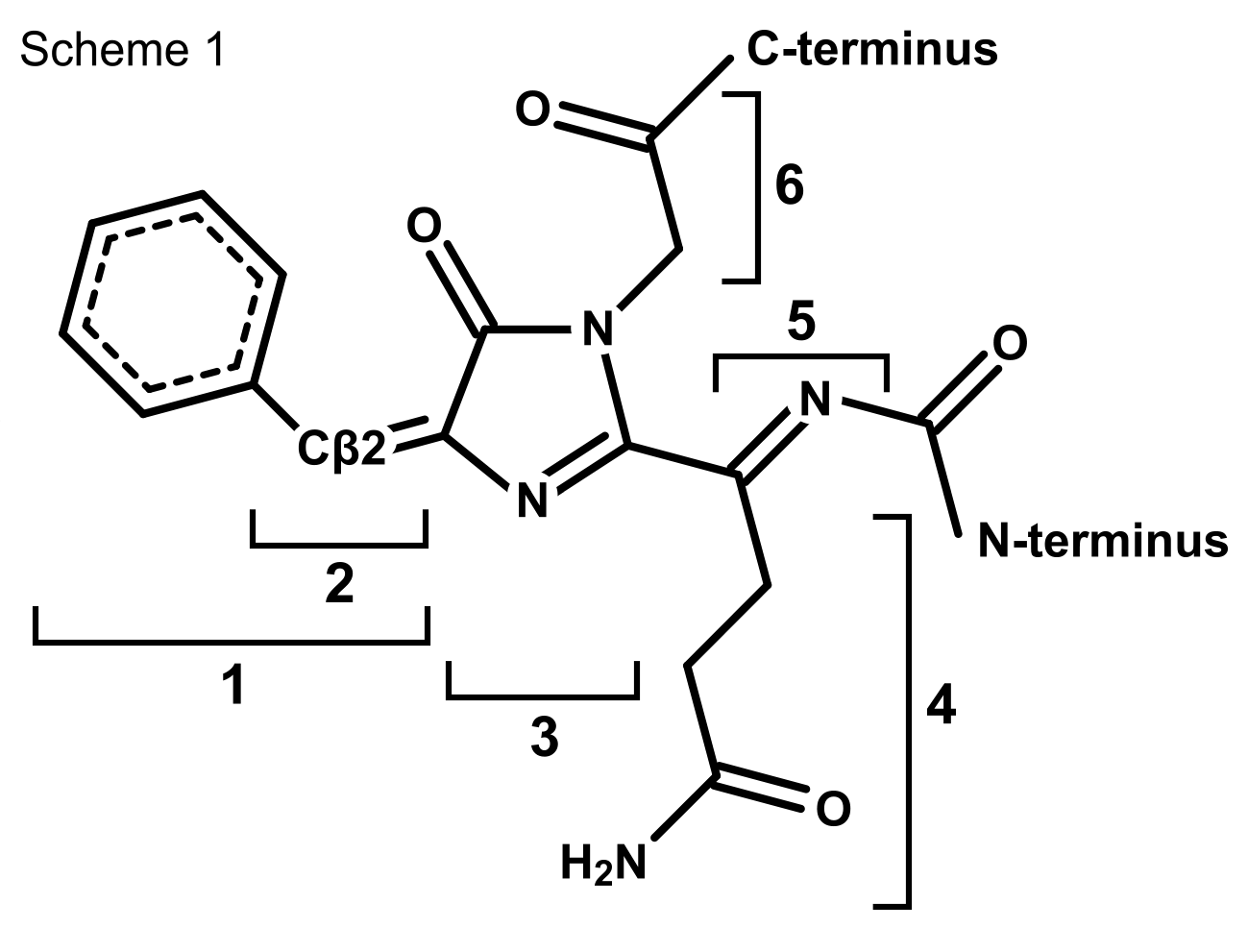

Supplement: Scheme S1 — (TIF) [file pone.0047331.s006.tif]

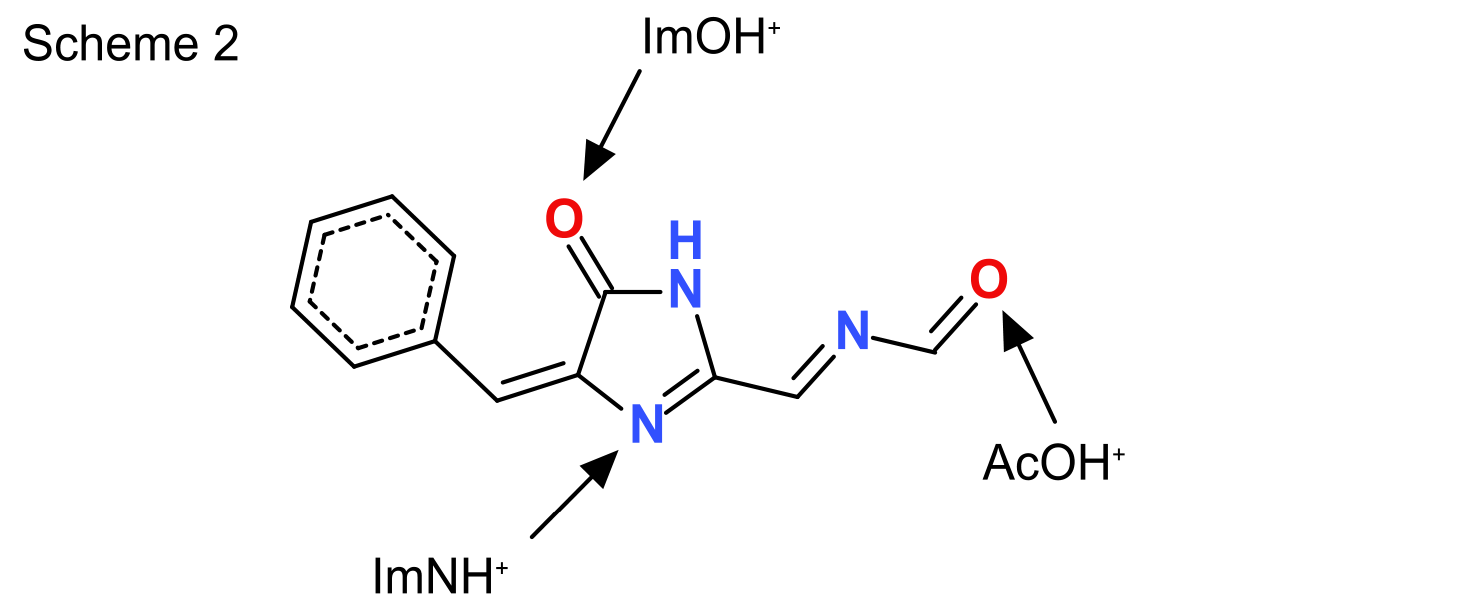

Supplement: Scheme S2 — (TIF) [file pone.0047331.s007.tif]
