# Supplementary material for: A Green Fluorescent Protein Containing a QFG Tri-Peptide Chromophore: Optical Properties and X-Ray Crystal Structure
Source: PLoS One. 2012 Oct 10;7(10):e47331. doi: 10.1371/journal.pone.0047331 (PMC3468514; doi:10.1371/journal.pone.0047331)
Supplement: Text S1 — (DOCX) [file pone.0047331.s005.docx]

**Supporting Information**

**Chemical quantum calculations** Although imidazolol tautomers have been suggested to play a role in the formation of FP chromophores [1], they have almost never been suggested to play a role in the photophysics of mature proteins; on the other hand, species with imidazolinone nitrogen protonation have been suggested [[2]](#_ENREF_13). The assignment of the 440nm band to an ImNH^+^-like tautomer is also supported by the lower ground state energy of the corresponding ImNH^+^ model, although the energy difference between this form and ImOH^+^ is not so large that it could not be overcome by the energy scales associated with intramolecular interactions in proteins [3]. Given this, assignment of the 440nm band to a nitrogen-protonated chromophore species seems more reasonable, although we cannot rule out the possibility of contribution by a chromophore protonated at the imidazolone oxygen.

The red shift of the absorbance of the singly protonated cations relative to the unprotonated state of the chromphore model seems, at first, to be contrary to expectations based on the well-documented halochromism of GFP chromophore models [4, 5]. However, it can be easily understood on the basis of the Brooker deviation rule [5] for the absorbance of cyanines and diarylmethane dyes represented by resonant Lewis structures. In the present case, we can apply the rule to the analysis of the hypothetical resonance outlined in Figure S4. The Brooker deviation rule states that there is a red limit to the absorbance of such dyes that is given by the average wavelength of the corresponding symmetric dyes generated by each of the terminal heterocycles. This limit is achieved when the different structures of the resonance have comparable energies. When the structures have very different energy, the energy of the asymmetric dye is shifted to the blue. The blue absorbance calculated for the Rtms5^Y66F^ chromophore is indicative that the neutral Lewis structure I (cf. Fig. S4) is lower in energy than the zwitterionic resonance structures IIa and IIb, which is entirely reasonable. Likewise, protonation of one of the available sites on the imidazolone or acylmine moieties would result in a significant lowering of the energies of the structures II, thereby leading to a red shift. Similar reasoning has previously been shown to apply in an analysis of the predicted excitation energies of GFP chromophore models [4].

**References**

1. Barondeau DJ, Tainer JA, Getzoff ED (2006) Structural evidence for an enolate intermediate in GFP fluorophore biosynthesis. J. Am. Chem. Soc. 128: 3166-3168.

2. Meech S (2009) Excited state reactions in fluorescent proteins. Chem. Soc. Rev. 38: 2922-2934.

3. Creighton, TE (1993) Proteins: Structures and Molecular Properties. New York, W.H. Freeman and Company. 507 p.

4. Dong J, Solntsev K, Tolbert LM (2006) Solvatochromism of the green fluorescence protein chromophore and its derivatives. J. Am. Chem. Soc. 128: 12038-12039.

5. Olsen, S (2010) A Modified Resonance-Theoretic Framework for Structure−Property Relationships in a Halochromic Oxonol Dye. J. Chem. Theory Comput. 6: 1089-1103.
